# Exosomes derived from human urine–derived stem cells ameliorate IL-1β-induced intervertebral disk degeneration

**DOI:** 10.1186/s12891-024-07636-2

**Published:** 2024-07-12

**Authors:** Guang Qian, Yueming Yu, Youhai Dong, Yang Hong, Minghai Wang

**Affiliations:** Department of Orthopedics, Shanghai Fifth People’s Hospital, Fudan University, No. 801, Heqing Road, Minhang District, Shanghai, 200240 China

**Keywords:** Apoptosis, Exosomes, Intervertebral disc degeneration

## Abstract

**Background:**

Human intervertebral disk degeneration (IVDD) is a sophisticated degenerative pathological process. A key cause of IVDD progression is nucleus pulposus cell (NPC) degeneration, which contributes to excessive endoplasmic reticulum stress in the intervertebral disk. However, the mechanisms underlying IVDD and NPC degeneration remain unclear.

**Methods:**

We used interleukin (IL)-1β stimulation to establish an NPC-degenerated IVDD model and investigated whether human urine–derived stem cell (USC) exosomes could prevent IL-1β-induced NPC degeneration using western blotting, quantitative real-time polymerase chain reaction, flow cytometry, and transcriptome sequencing techniques.

**Results:**

We successfully extracted and identified USCs and exosomes from human urine. IL-1β substantially downregulated NPC viability and induced NPC degeneration while modulating the expression of SOX-9, collagen II, and aggrecan. Exosomes from USCs could rescue IL-1β-induced NPC degeneration and restore the expression levels of SOX-9, collagen II, and aggrecan.

**Conclusions:**

USC-derived exosomes can prevent NPCs from degeneration following IL-1β stimulation. This finding can aid the development of a potential treatment strategy for IVDD.

**Supplementary Information:**

The online version contains supplementary material available at 10.1186/s12891-024-07636-2.

## Background

Intervertebral disk (IVD) degeneration (IVDD) is the most common cause of lower-back pain and exerts a high burden on global healthcare systems. IVDD is a complex and incompletely understood degenerative pathological process that is related to gene expression, physical stress, cellular senescence, and functional structure changes [1–3].

The inner nucleus pulposus cell (NPC) and outer annulus fibrosus (AF) are important components of the IVD. Only the outermost layers of the AF contain blood vessels. The NPCs diffuse nutrients from capillary buds in the cartilaginous endplate to fulfill metabolic activities. Therefore, NPCs are highly likely to be affected by limited vascular and nutritional supply in the IVD. Conversely, the extracellular matrix (ECM) is maintained by the NPC. IVDD progression is initiated and promoted via NPC and ECM degradation. Thus, rescuing NPC degeneration may be an effective method of obstructing or reversing IVDD.

Currently, to the best of our knowledge, there is no available approved treatment to effectively prevent the progressive degeneration of the IVD [4, 5]. Widely accepted therapies for IVDD include pharmacological and surgical interventions. However, these interventions cannot retard or restore the progression of the disease or repair the functionality of the IVD. Stem cell–based therapies have demonstrated several promising effects in the regeneration of the IVD, with varying degrees of effectiveness in reversing disk degeneration in the long term [6]. However, biological and ethical limitations constitute the main barriers, presenting significant challenges to stem cell–based techniques [7, 8].

Common IVDD treatment focuses on restoring degenerative NPCs, and urine-derived stem cells (USCs) are a possible medium for treatment. USC transplantation may reduce IVDD progression partly by rescuing degenerative NPCs or attenuating excessive cell injuries during IVDD. Previous studies have reported the functional role of USCs in reducing NPC degeneration and maintaining NPC proliferation [9]. Exosomes are important mediators of intercellular communication; particularly, stem cell–derived exosomes can reportedly maintain the viability of the origin cells without additional risks [4, 10, 11]. USC-derived exosomes contain various regulatory signaling cytokines, proteins, lipids, and nucleic acids, which play crucial roles in USC-based cell therapy. Exosomal transplantation can regulate proliferation, differentiation, apoptosis, and many other cellular activities. However, whether and how human USC exosomes prevent interleukin (IL)-1β-induced IVDD remain largely unknown.

Herein, we extracted and cultured USCs and established an NPC degeneration model using IL-1β stimulation. Cell viability assays, immunohistochemistry, western blotting, quantitative real-time polymerase chain reaction (q-PCR), and flow cytometry techniques were employed to examine the effects of USCs or exosomes on degradative NPCs. In addition, we performed whole-transcriptome sequencing (circRNA, lncRNA, miRNA, and mRNA) and functional pathway enrichment to determine the mechanisms by which USC exosomes prevent IVDD. The elucidation of these mechanisms can help develop a potential clinical strategy for treating IVDD.

## Methods

### Extraction and identification of human urine–derived stem cells

The study was approved by the Ethics Committee of Shanghai Fifth People’s Hospital, Fudan University (2022) Lun Shen No. (085). Written informed consent was obtained from the participants. The culture of human USCs followed a previously described procedure [12]. In brief, clean midstream urine (200 mL) was obtained from three healthy adults. The urine was centrifuged at 400 × *g* for 10 min, and the supernatant was discarded. The pellet was resuspended in phosphate-buffered saline, centrifuged at 200 × *g* for 10 min, and resuspended with USC primary medium (3 mL, 10% fetal bovine serum, 1% penicillin–streptomycin double antibody, Renal Epithelial Cell Growth Medium (REBM) mixed with Dulbecco’s Modified Eagle Medium (DMEM)/F-12 in a ratio of 1:1). USCs were cultured at 37 °C and 5% CO_2_. On day 3, the primary medium was changed to USC complete medium (10% fetal bovine serum without exosomes, 1% penicillin–streptomycin antibody, 1% GlutaMAX, 1% NEAA, PDGF-BB 5 ng/mL, FGF-b 5 ng/mL, EGF 5 ng/mL, DMEM/F-12 and REBM in a ratio of 1:1). The identification of USCs was verified by expression of the stem cell–positive markers, CD29, CD44, CD73, and CD90, and the stem cell–negative markers, CD34 and CD45.

### Extraction and identification of exosomes from human USCs

The USC medium was collected and centrifuged at 300 × *g* (4 °C, 10 min). The supernatant was then collected and centrifuged at 2,000 × *g* (first cycle, 4 °C, 10 min) and 10,000 × *g* (second cycle, 4 °C, 30 min). The final supernatant was centrifuged at 100,000 × *g* (4 °C, 70 min), and the precipitate contained the exosomes. The morphology, particle sizes, and CD9, CD63, or TSG101 surface markers of the extracted exosomes were detected by transmission electron microscopy, nanoparticle tracking analysis, and western blotting, respectively, to confirm successful exosome extraction.

### Extraction and culture of human IVD NPCs

Normal IVD nucleus pulposus tissues were collected from patients who underwent decompression through the lateral anterior approach for thoracolumbar burst fractures in the Department of Orthopedics of our hospital. The patients underwent routine magnetic resonance examination before surgery, and the degree of IVD degeneration was determined according to the Pfirrmann magnetic resonance score. Under sterile conditions, intraoperative disk tissue was collected for cell isolation in the laboratory. The isolated NPCs were cultured at 37 °C and 5% CO_2_ according to the protocol described previously [12], and the subsequent experiments were performed when the cells had been passaged three times.

### IL-1β-induced NPC degeneration model

Asporin is an ECM protein that activates the p65 pathway in human NPCs. Several studies have reported that asporin levels considerably increase in degenerated NPCs of patients with IVDD. IL-1β can upregulate the asporin expression level. Herein, we used IL-1β stimulation to create an NPC degeneration model [13]. Normal NPCs were cultured with IL-1β (10 ng/mL) for 24 h to establish IL-1β-induced degenerated NPCs [13]. Normal NPCs were cocultured with IL-1β (10 ng/mL) and human USCs or USC-derived exosomes (10 μg/mL). In addition, GW4869 (10 μg/mL) was added to IL-1β-treated NPCs cocultured with USCs to inhibit exosome secretion from the USCs.

### CCK-8 cell proliferation and cytotoxicity assay

The proliferation activity of NPCs was detected using a CCK-8 cell proliferation and cytotoxicity assay kit (BBI Life Sciences, E606335-0500). The protocols are described in the manufacturer’s instructions [14, 15]. Briefly, cells were seeded in a 96-well plate at 5,000/cm^2^ and cultured for 6 h at 37 °C and 5% CO_2_. Then, 10 μL of CCK-8 solution was added, and the cells were incubated for an additional 2 h. The absorbance value was measured at 450 nm using a microplate reader to determine cell proliferation.

### Beta-gal staining assay

Cultured cells were stained using the Senescence β-Galactosidase Staining Kit (Beyotime, C0602) according to the manufacturer’s instructions. Briefly, the cells were washed once with Dulbecco’s Phosphate-Buffered Saline, fixed, and then stained with the reagents provided in the kit. Data were obtained in triplicate.

### Flow cytometry

NPC apoptosis was determined via flow cytometry using annexin V/PI cell apoptosis assays. The protocols were conducted as described previously [16–18]. The cells were digested with trypsin without ethylenediaminetetraacetic acid and collected after centrifugation at 300 × *g* at 4 °C for 5 min. The staining was performed according to the instructions given in the AnnexinV /PI cell apoptosis detection kit, and flow cytometry was performed within 1 h to determine cell apoptosis.

### Western blotting

Western blotting analysis was performed following the procedures previously described [19, 20]. In brief, USCs or NPCs were lysed in RIPA buffer containing 1% phenylmethanesulfonyl fluoride (PMSF). Protein concentrations were determined using a standard bicinchoninic acid (BCA) assay kit. Target proteins were resolved via sodium dodecyl sulfate–polyacrylamide gel electrophoresis. After gel electrophoresis, the membrane was blocked with 5% skim milk. The membrane was incubated with primary antibodies against SOX-9, collagen II, aggrecan, and GAPDH at 4 °C overnight. The membrane was then incubated with HRP-labeled secondary antibodies, and chemiluminescence images were obtained using an automatic optimized exposure instrument.

### Quantitative real-time polymerase chain reaction

Total RNA was extracted from NPCs and USCs using the TaKaRa MiniBEST Universal RNA Extraction Kit (Takara, Japan). q-PCR was performed using the PrimeScript™ RT Reagent Kit with gDNA Eraser (Takara, Japan), and mRNA was measured using TB Green^®^ Premix Ex Taq™ II (Takara, Japan). q-PCR assays were performed on a QuantStudio 3 Real-Time PCR system (Thermo Fisher Scientific, USA). The relative mRNA levels of SOX-9, collagen II, and aggrecan were normalized from the Ct values of GAPDH according to the 2^−ΔΔ*CT*^ calculation method [21].

### RNA extraction, library preparation, and transcriptome sequencing

Three groups of cells were collected for transcriptome sequencing, with three biological replicates per group: IL-1β samples, USCs + IL-1β samples, and exosomes + IL-1β samples. The sequencing procedures were conducted as previously described [22, 23]. The RNA was extracted using a Trizol kit. The cDNA library construction for these samples was performed using the Illumina TruSeq RNA Sample Preparation Kit, and whole-transcriptome sequencing was performed on the Illumina platform to obtain the raw RNA-seq data of the cicRNA, lncRNA, miRNA, and mRNA expression profiles. Gene expression values were estimated from RNA-seq data from tumor samples using Tophat2 (Tophat 2 v2.1.0 [http://ccb.jhu.edu/software/tophat/downloads/tophat-2.1.0.tar.gz]). The paired-end transcriptome sequencing reads were aligned with the human reference genome (GRCh37/hg19) in Tophat2 [24].

### Analysis of differentially expressed RNA (cicRNA, lncRNA, miRNA, and mRNA)

We used the R package DEseq2 to compare expression differences between the different cell groups, and paired tests were performed [24]. We [25] used a fold change of > 2.0 and a false discovery rate of < 0.05 as thresholds to define differentially expressed genes (DEGs).

### Functional enrichment analysis of hub genes in ceRNA networks

To better understand the functions of related circRNAs and lncRNAs in the ceRNA networks, DAVID online software was used to analyze the Gene Ontology (GO) and Kyoto Encyclopedia of Genes and Genomes signaling pathway enrichment of circRNAs or lncRNAs-targeted mRNAs to determine the biological functions that exosomes might affect via the ceRNA network signaling pathway.

### Statistical analysis

All data were analyzed using SPSS 20.0 software. All experiments were repeated at least three times and the measurement data were expressed as the mean ± SD. One-way analysis of variance (ANOVA) or *t*-test was used to identify significant differences between the different groups, and a *P* value of < 0.05 was considered statistically significant.

## Results

### IL-1β-induced human IVD NPC degeneration

Inflammatory cytokines, including IL-1β, IL-6, and tumor necrosis factor-α (TNF-α), are closely related to IVDD [13]. Herein, we used IL-1β stimulation to create an NPC degeneration model. IL-1β upregulated asporin expression by activating the p65 pathway in human NPCs.

**Fig. 1:**
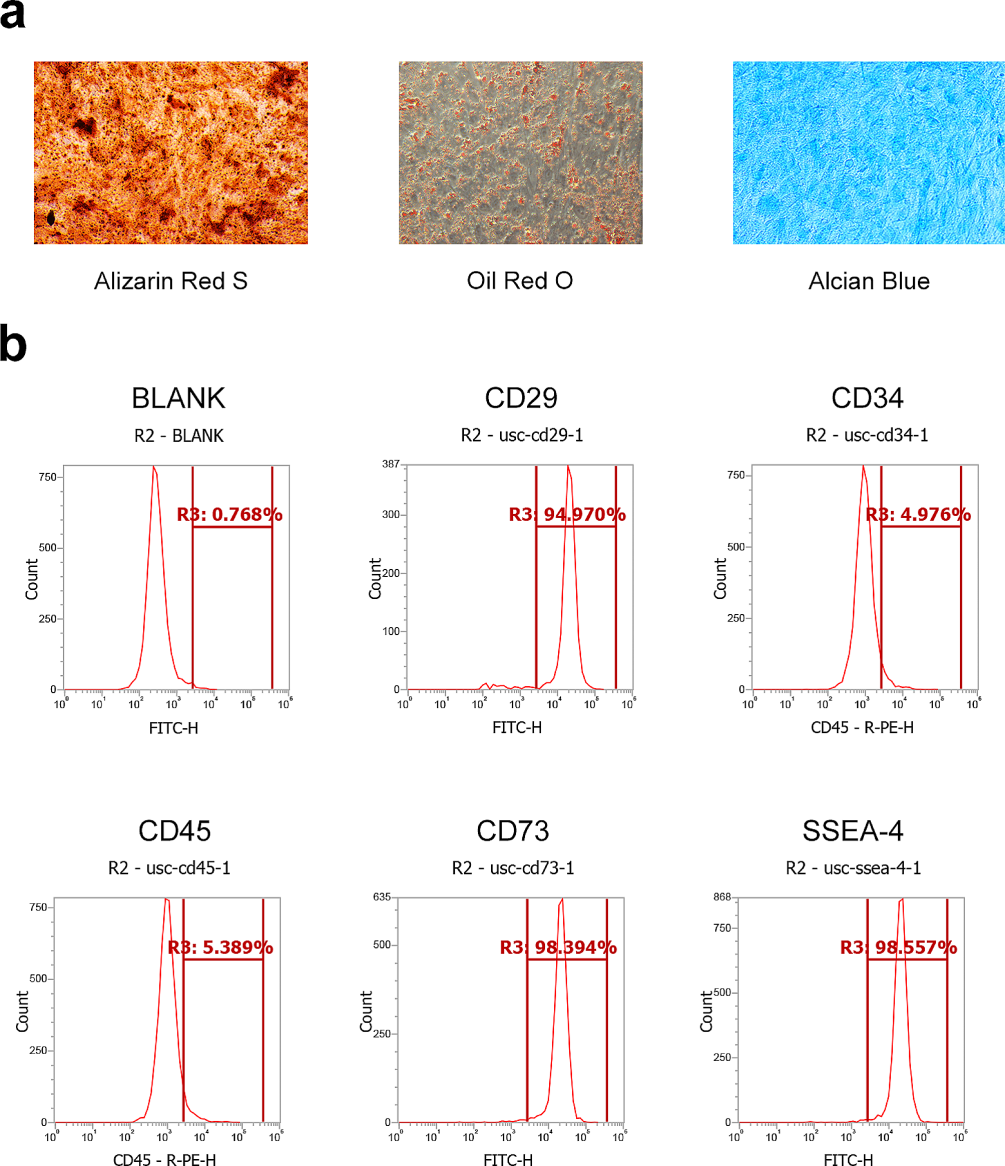
Identification of human urine–derived stem cells. (**a**) Representative images of alizarin red S-, alcian blue-, and oil red O-stained human USCs. (**b**) Cell-surface antigen detection of CD29, CD34, CD45, CD73, and SSEA-4 in human USCs

We successfully extracted stem cells from human urine. To identify the cell purity of the extracted USCs, alizarin red S, alcian blue, and oil red O staining was performed (Fig. 1a). In addition, the surface antigen expression levels of CD29, CD34, CD45, CD73, and SSEA-4 in human USCs were examined (Fig. 1b).

**Fig. 2:**
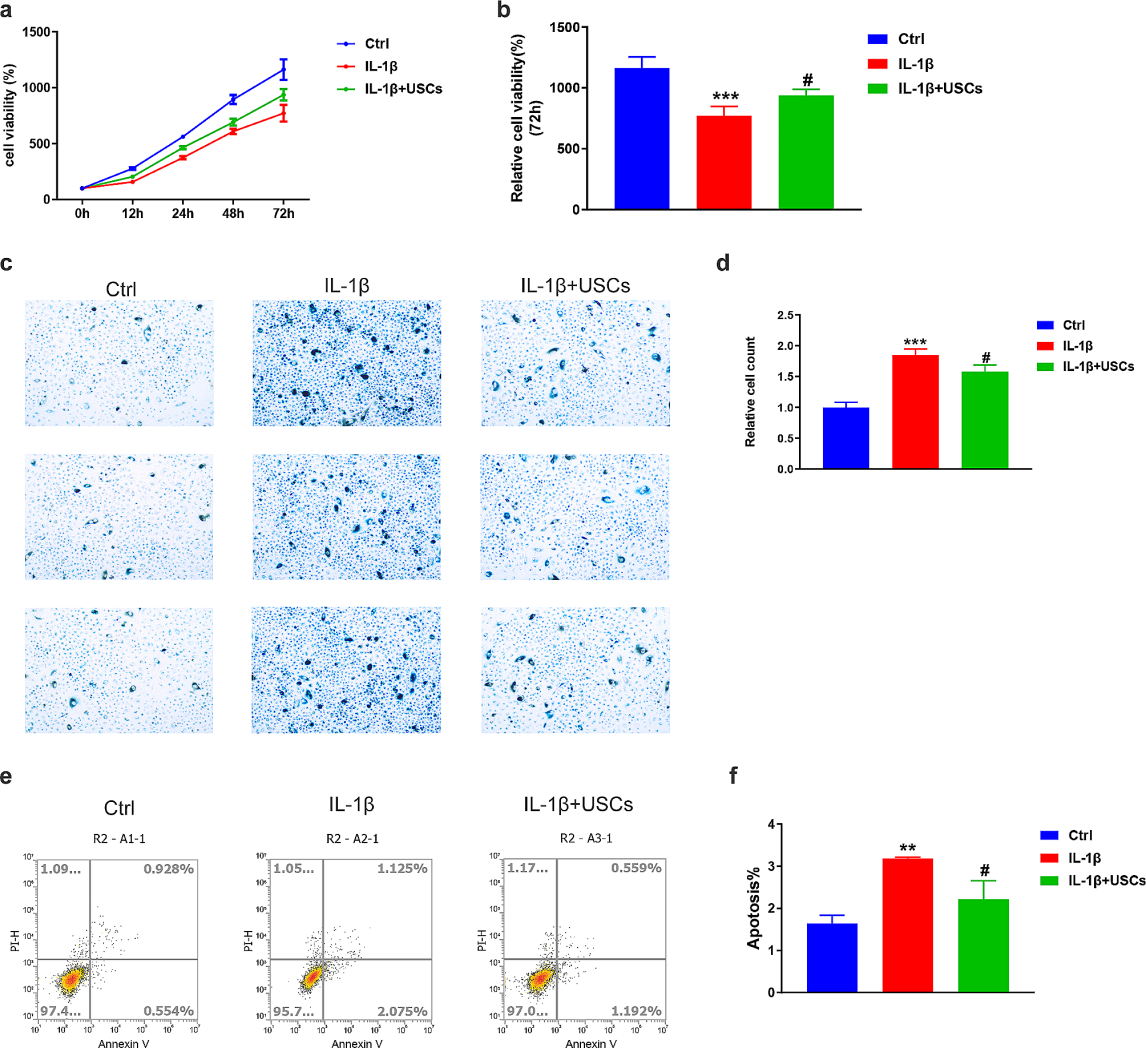
IL-1β-induced human intervertebral disk nucleus pulposus (NPC) degeneration in vitro. (**a**) Time-coursed curves illustrating NPC viability rates at 0–72 h in CCK8 assays. IL-1β stimulation induced NPC degeneration while USCs ameliorated the effects of IL-1β on NPCs. Blue indicates the control group, red indicates the IL-1β group, and green indicates the IL-1β + USCs group. (**b**) Bar plot representing the relative cell viability rates at 72 h, indicating that IL-1β significantly upregulated NPC degeneration. *** *P* = 0.000, # *P* = 0.045. One-way analysis of variance (ANOVA) with Bonferroni’s post-hoc test. (**c**, **d**) NPC beta-gal staining assays to detect cell apoptosis following IL-1β stimulation (**c**) and bar plots indicating that IL-1β promoted NPC degeneration (**d**). *** *P* = 0.005, # *P* = 0.016. One-way ANOVA with Bonferroni’s post-hoc test. (**e**, **f**) Representative gating strategy for identifying annexin V−/PI + NPCs, in which annexin V−/PI or annexin V+/PI + indicates live NPCs and apoptotic NPCs, respectively (**e**). Bar plots summarizing the flow cytometry results. IL-1β stimulation induced annexin V−/PI to annexin V+/PI + transfer in NPCs (**f**). ** *P* = 0.000, #*P* = 0.024. One-way ANOVA with Bonferroni’s post-hoc test

To investigate the effects of USCs on degenerative NPCs, we established a degenerative NPC model via IL-1β stimulation following previously described protocols. CCK8 assays were performed to detect the effects of IL-1β on NPC cell viability. The results revealed that IL-1β significantly downregulated NPC viability in vitro compared with the control. The combined stimulation of IL-1β and USCs gently upregulated NPC viability compared with the IL-1β group (Fig. 2a and b). Furthermore, beta-gal staining and flow cytometry assays confirmed similar results, indicating that IL-1β could induce NPC degeneration and USCs exerted protective effects on NPC viability (Fig. 2c and f). Thus, we conducted subsequent investigations using this IL-1β-induced NPC degeneration model.

### IL-1β induced NPC degeneration by modulating the expression of SOX-9, collagen II, and aggrecan in human IVD NPCs

SOX-9 is a necessary factor for cartilage development and an important transcriptional factor for chondrogenesis, which is required in chondrocyte differentiation [22]. Collagen II is a subunit of the fibrous proteins that constitute the ECM, which provides a highly compatible environment [26, 27]. Aggrecan is a cartilage-specific proteoglycan core protein and is an integral part of the ECM in cartilaginous tissue [28].

IL-1β-induced NPC degeneration led to chondrocyte degeneration. Because chondrocyte degeneration is closely related to anabolic genes, we evaluated the levels of three biomarkers in NPCs following IL-1β stimulation: SOX-9, collagen II, and aggrecan.

**Fig. 3:**
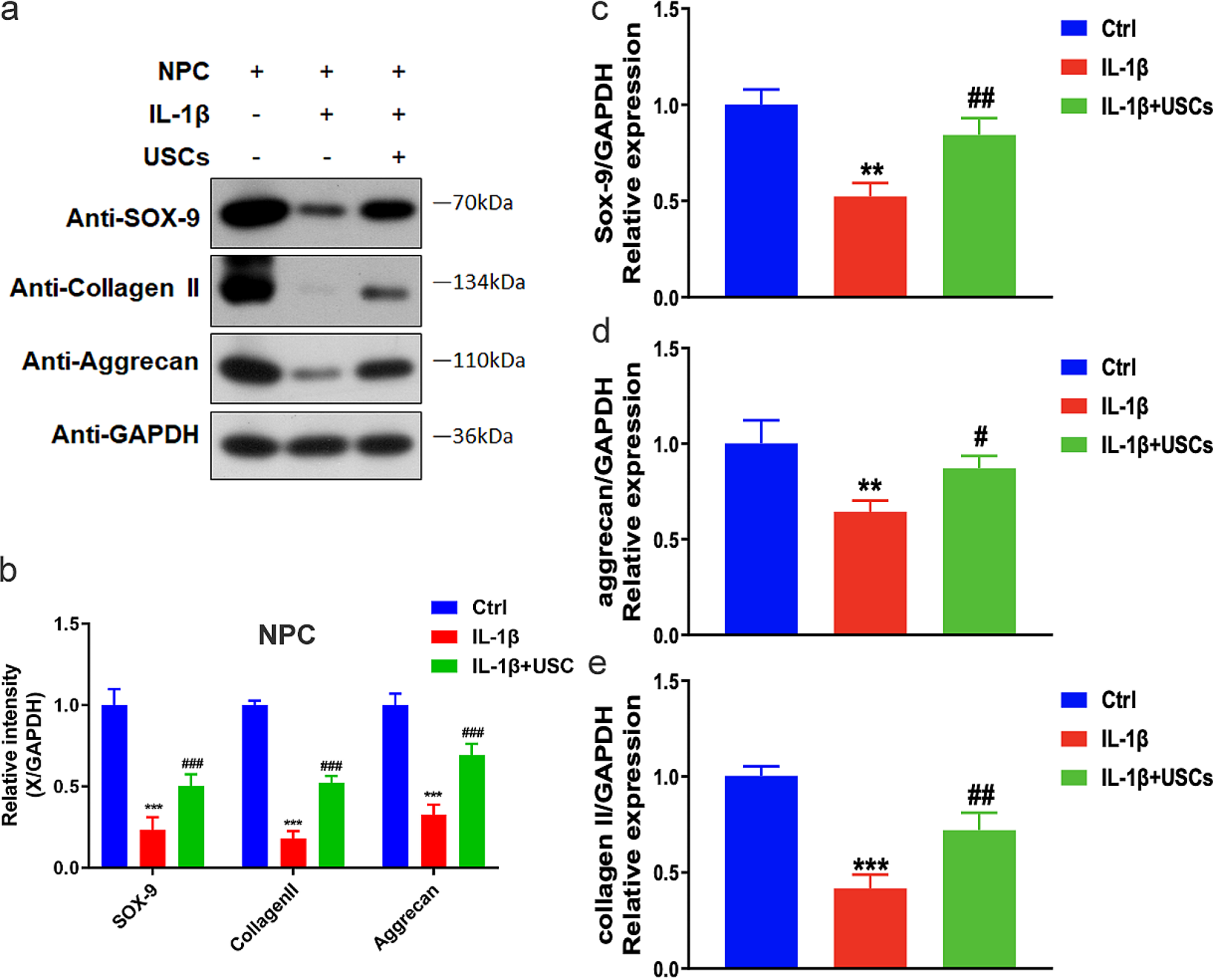
IL-1β induced NPC degeneration by modulating the expression levels of SOX-9, collagen II, and aggrecan in human intervertebral disk NPCs. (**a**) Representative western blotting results showing the expression changes in SOX-9, collagen II, and aggrecan after stimulation with IL-1β or USCs. (**b**) Comparison of average relative densitometric quantifications of the immunoreactive bands of SOX-9, collagen II, and aggrecan. ** *P* = 0.003, ## *P* = 0.005, ### *P* = 0.000. One-way ANOVA with Bonferroni’s post-hoc test. (**c**–**e**) Bar plots showing the relative mRNA levels of SOX-9, collagen II, and aggrecan. ** *P* = 0.001, ## *P* = 0.000, ### *P* = 0.003. One-way ANOVA with Bonferroni’s post-hoc test

These biomarkers can indicate the relative level of NPC viability. Western blotting revealed that IL-1β downregulated the expression levels of SOX-9, collagen II, and aggrecan (Fig. 3a and b), and q-PCR data (Fig. 3c and e) confirmed similar results for mRNA levels. The expression levels in the IL-1β + USCs group were slightly higher compared with those in the IL-1β group (Fig. 2b and e). Therefore, our experimental results revealed that chondrocyte degeneration was involved in IL-1β-induced NPC degeneration and that USCs could hinder NPC degeneration in vitro.

### USCs could rescue IL-1β-induced NPC degeneration through exosomes

**Fig. 4:**
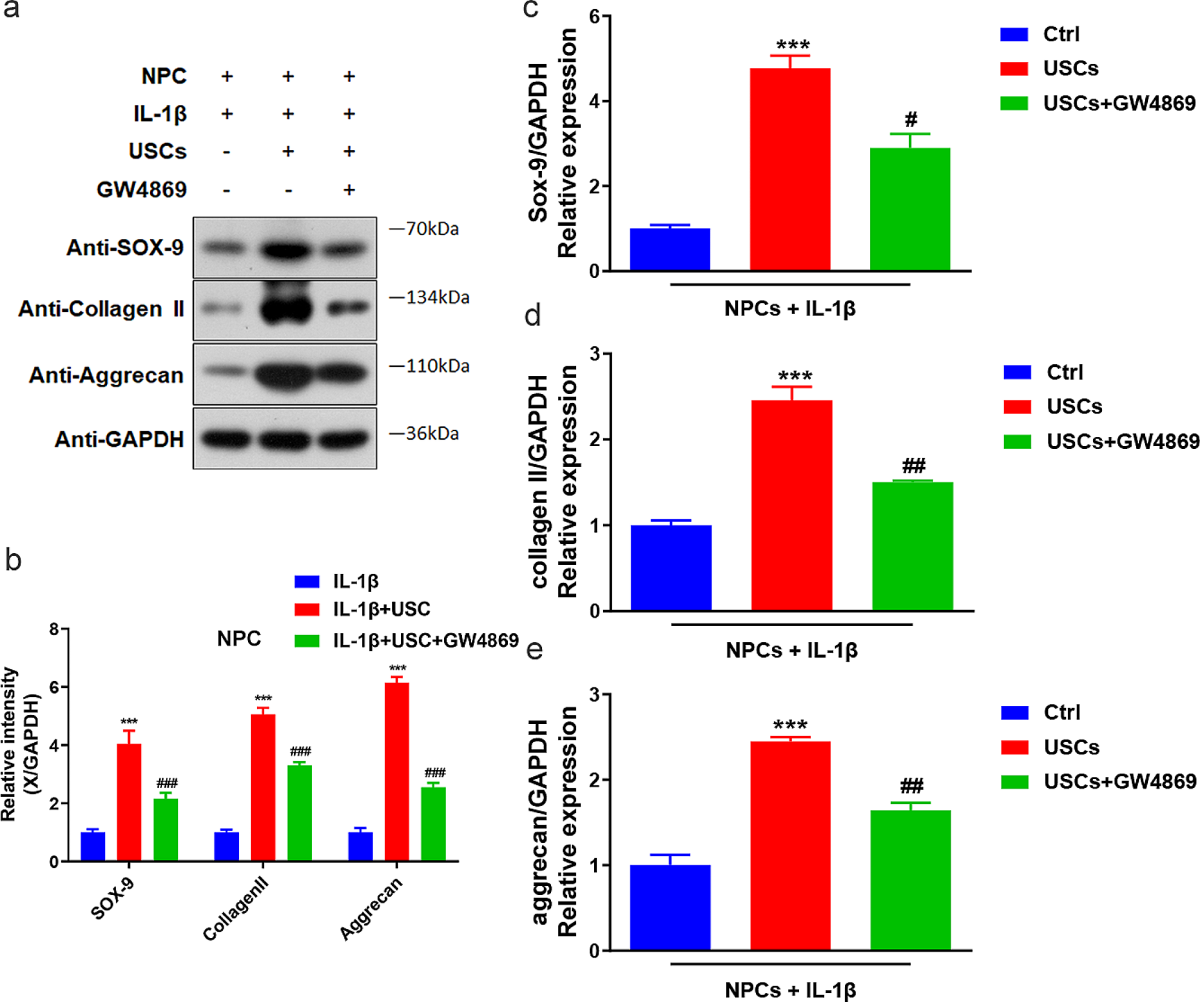
Dynamic expression level changes in SOX-9, collagen II, and aggrecan in human intervertebral disk NPCs following USC intervention. (**a**) Representative western blotting results showing the expression changes in SOX-9, collagen II, and aggrecan with the intervention of USCs or USCs + GW4869. (**b**) Comparison of average relative densitometric quantifications of the immunoreactive bands of SOX-9, collagen II, and aggrecan. ** *P* = 0.003, ## *P* = 0.001, ### *P* = 0.000. One-way ANOVA with Bonferroni’s post-hoc test. (c–e) Bar plots showing the relative mRNA levels of SOX-9, collagen II, and aggrecan. ** *P* = 0.005, ## *P* = 0.001, ### *P* = 0.000. One-way ANOVA with Bonferroni’s post-hoc test. GW4869: an exosome-specific inhibitor

**Fig. 5:**
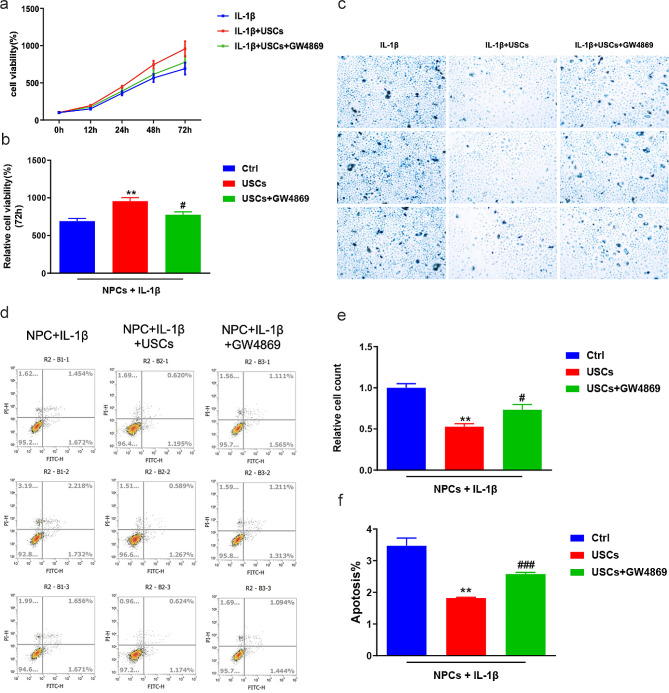
USCs could rescue IL-1β-induced NPC degeneration through exosomes in vitro. (**a**, **b**) Time-coursed curves illustrating NPC viability rates at 0–72 h in CCK8 assays. Blue represents the IL-1β group, red represents the IL-1β + USCs group, and green represents the IL-1β + USCs + GW4869 group (**a**). Bar plot summarizing the cell viability rate at 72 h, indicating that USCs could rescue IL-1β-induced NPC degeneration in vitro (**b**). *** *P* = 0.005, # *P* = 0.04. One-way ANOVA with Bonferroni’s post-hoc test. (**c**, **e**) NPC beta-gal staining assays to detect cell apoptosis following IL-1β stimulation. (**c**) Bar plots indicating that IL-1β promoted NPC apoptosis (**e**). ** *P* = 0.003, # *P* = 0.001. One-way ANOVA with Bonferroni’s post-hoc test. (**d**, **f**) Representative gating strategy for identifying annexin V+/PI + NPCs, in which annexin V−/PI or annexin V+/PI + represent live NPCs or apoptotic NPCs, respectively (**d**). Bar plots summarizing the flow cytometry results, indicating that IL-1β stimulation induced annexin V−/PI to annexin V+/PI + transfer in NPCs (**f**). ** *P* = 0.008, # *P* = 0.003. One-way ANOVA with Bonferroni’s post-hoc test. GW4869: an exosome-specific inhibitor

The previous results illustrated the protective effects of USCs on NPCs. Subsequently, we examined whether USCs rescued IL-1β-induced NPC degeneration through exosomes. We used the exosome-specific inhibitor GW4869 to suppress exosome functions. Western blotting revealed that GW4869 could downregulate the IL-1β-induced elevated expression levels of SOX-9, collagen II, and aggrecan (Fig. 4). The q-PCR assays demonstrated similar results. Thus, our results showed that USCs could rescue IL-1β-induced NPC degeneration through exosomes. In addition, CCK8 (Fig. 5a and b), beta-gal staining (Fig. 5c and e), and flow cytometry (Fig. 5d and f) assays were performed to examine the effects of exosomes on NPC degeneration. We found that GW4869 could suppress the protective effects of USCs on NPC degeneration, indicating that exosomes were involved in the USC-mediated rescue of IL-1β-induced NPC degeneration.

### Exosomes facilitate USCs to prevent NPC degeneration

**Fig. 6:**
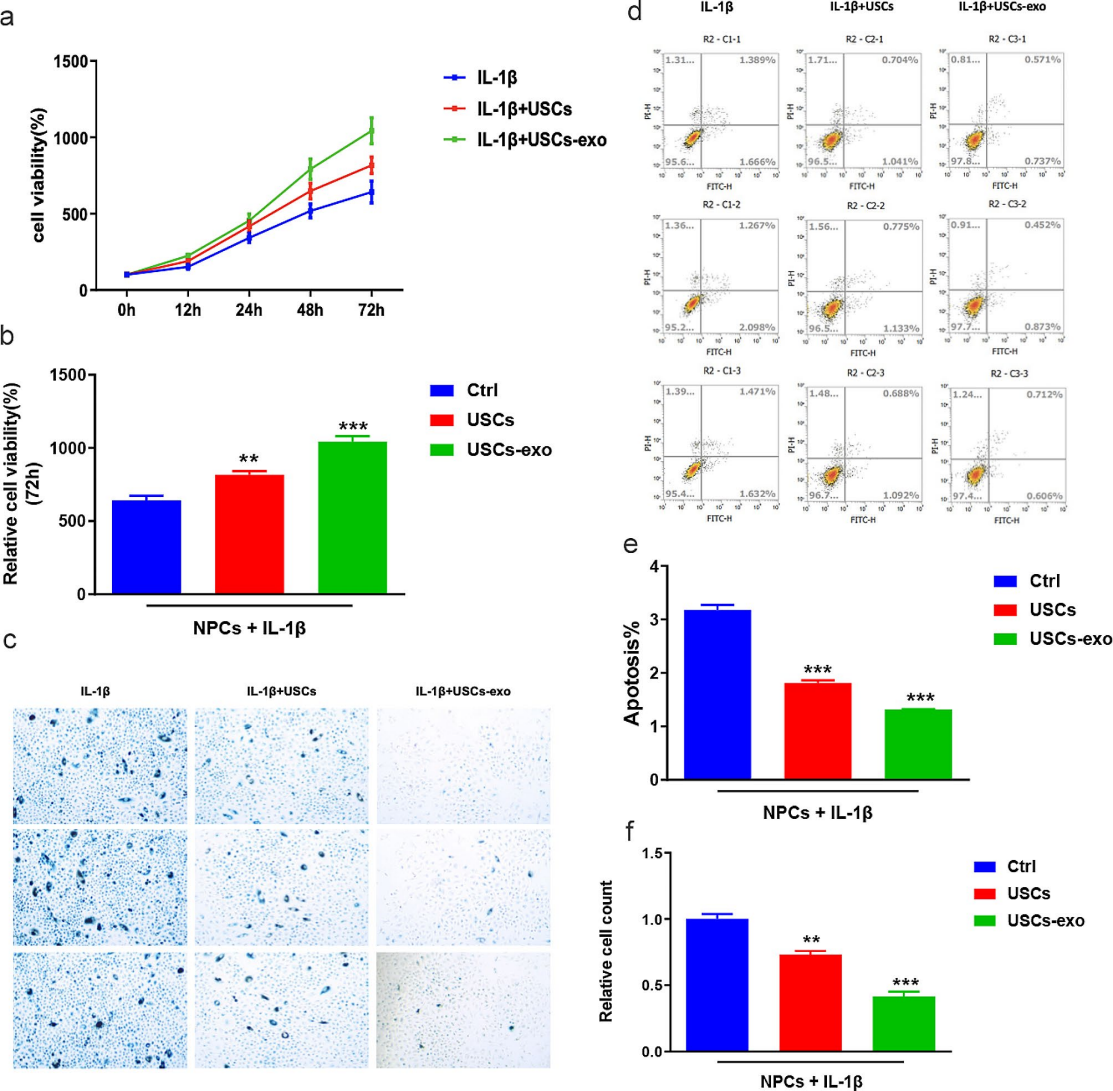
Exosomes enhanced the rescue effects of USCs on IL-1β-induced NPC degeneration in vitro. (**a**, **b**) Time-coursed curves illustrating NPC viability rates at 0–72 h in CCK8 assays. Blue represents the IL-1β group, red represents the IL-1β + USCs group, and green represents the IL-1β + USCs + exo group (**a**). Bar plot summarizing the cell viability rate at 72 h, indicating that USCs could rescue IL-1β-induced NPC degeneration in vitro (**b**). *** *P* = 0.03, # *P* = 0.007. One-way ANOVA with Bonferroni’s post-hoc test. (**c**, **e**) Representative images of NPC beta-gal staining assays with IL-1β or IL-1β + exo stimulation (**c**) and bar plots showing that exosomes could enhance the rescue effects of USCs on IL-1β-induced NPC degeneration (**e**). *** *P* = 0.01, # *P* = 0.005. One-way ANOVA with Bonferroni’s post-hoc test. (**d**, **f**) Representative gating strategy for identifying annexin V+/PI + NPCs, in which annexin V−/PI or annexin V+/PI + represent live NPCs or apoptotic NPCs, respectively (**d**). Bar plots summarizing the flow cytometry results, indicating that USCs or USCs + exo could ameliorate NPC degeneration. (**f**). ** *P* = 0.02, # *P* = 0.000. One-way ANOVA with Bonferroni’s post-hoc test. GW4869: an exosome-specific inhibitor

**Fig. 7:**
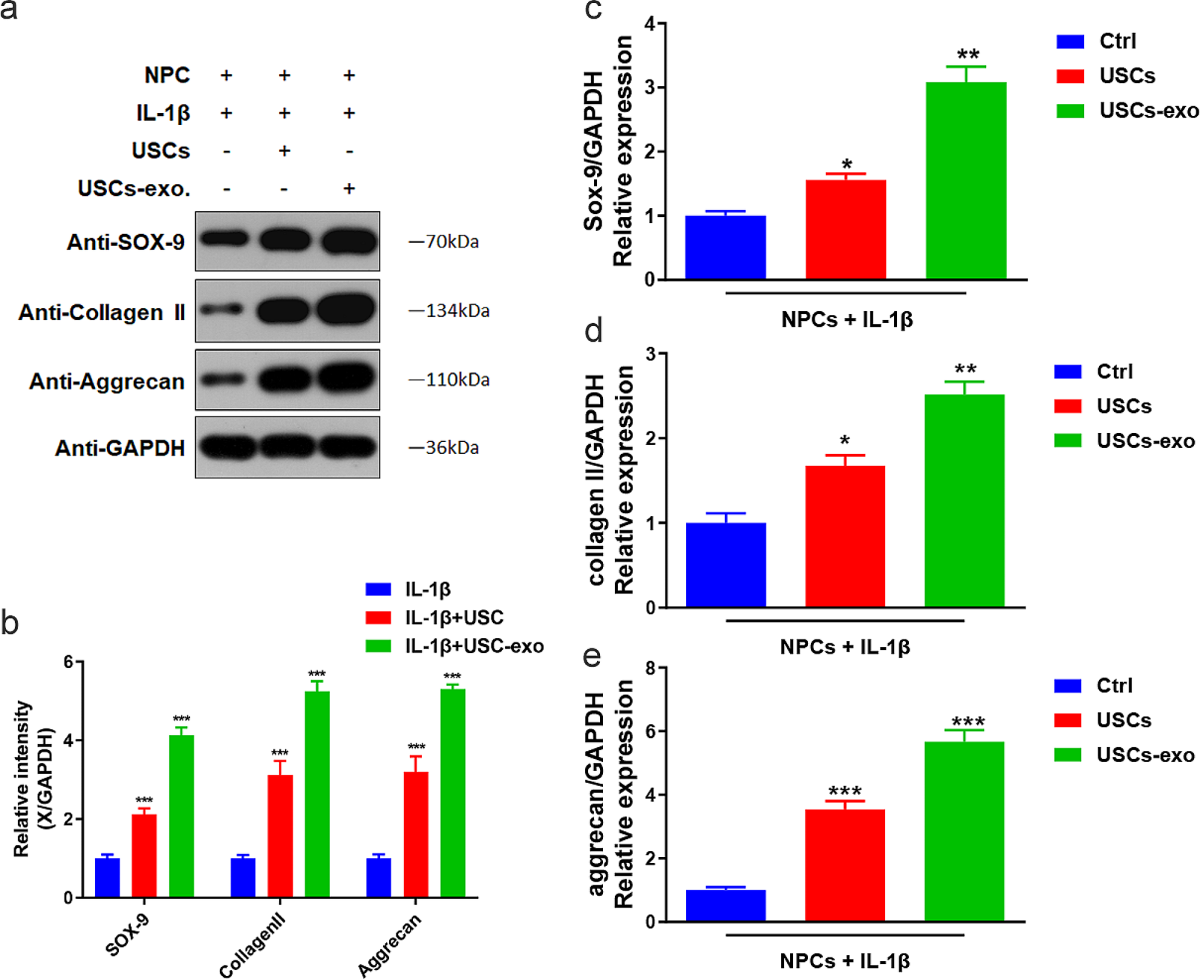
Exosomes facilitated USCs to upregulate the expression levels of SOX-9, collagen II, and aggrecan in human intervertebral disk NPC following IL-1β stimulation. (**a**) Representative western blotting results showing the expression changes in SOX-9, collagen II, and aggrecan with the intervention of USCs or USCs + exo in IL-1β-induced dNPCs. (**b**) Comparison of the average relative densitometric quantifications of the immunoreactive bands of SOX-9, collagen II, and aggrecan. ** *P* = 0.002, ## *P* = 0.005, ### *P* = 0.000. One-way ANOVA with Bonferroni’s post-hoc test. (**c**–**e**) Bar plots showing the relative mRNA levels of SOX-9, collagen II, and aggrecan. ** *P* = 0.01, ## *P* = 0.03, ### *P* = 0.001. One-way ANOVA with Bonferroni’s post-hoc test. GW4869: an exosome-specific inhibitor

The extraction of exosomes from USCs revealed that USCs rescued IL-1β-induced NPC degeneration through exosomes (Fig. 6). Subsequently, we examined whether exosomes from USCs (USCs-exo) could independently protect NPCs from degeneration following IL-1β stimulation. Referring to the previous experiments, we investigated the effects of USCs-exo on SOX-9, collagen II, and aggrecan expression levels and NPC viability and apoptosis. Our results revealed that USCs-exo significantly upregulated the expression levels of SOX-9, collagen II, and aggrecan compared with USCs alone (Fig. 7). Furthermore, USCs-exo protected NPCs from apoptosis and maintained NPC cell viability under IL-1β stimulation more effectively compared with USCs alone. Therefore, we concluded that exosomes facilitate the USC-mediated prevention of NPC degeneration and play a vital role in the process of NPC degeneration. Thus, USCs-exo can be used as a clinical resource for IVDD treatment in the near future [29].

### Whole-transcriptome sequencing analysis revealed an enriched pathway for NPC degeneration

**Fig. 8:**
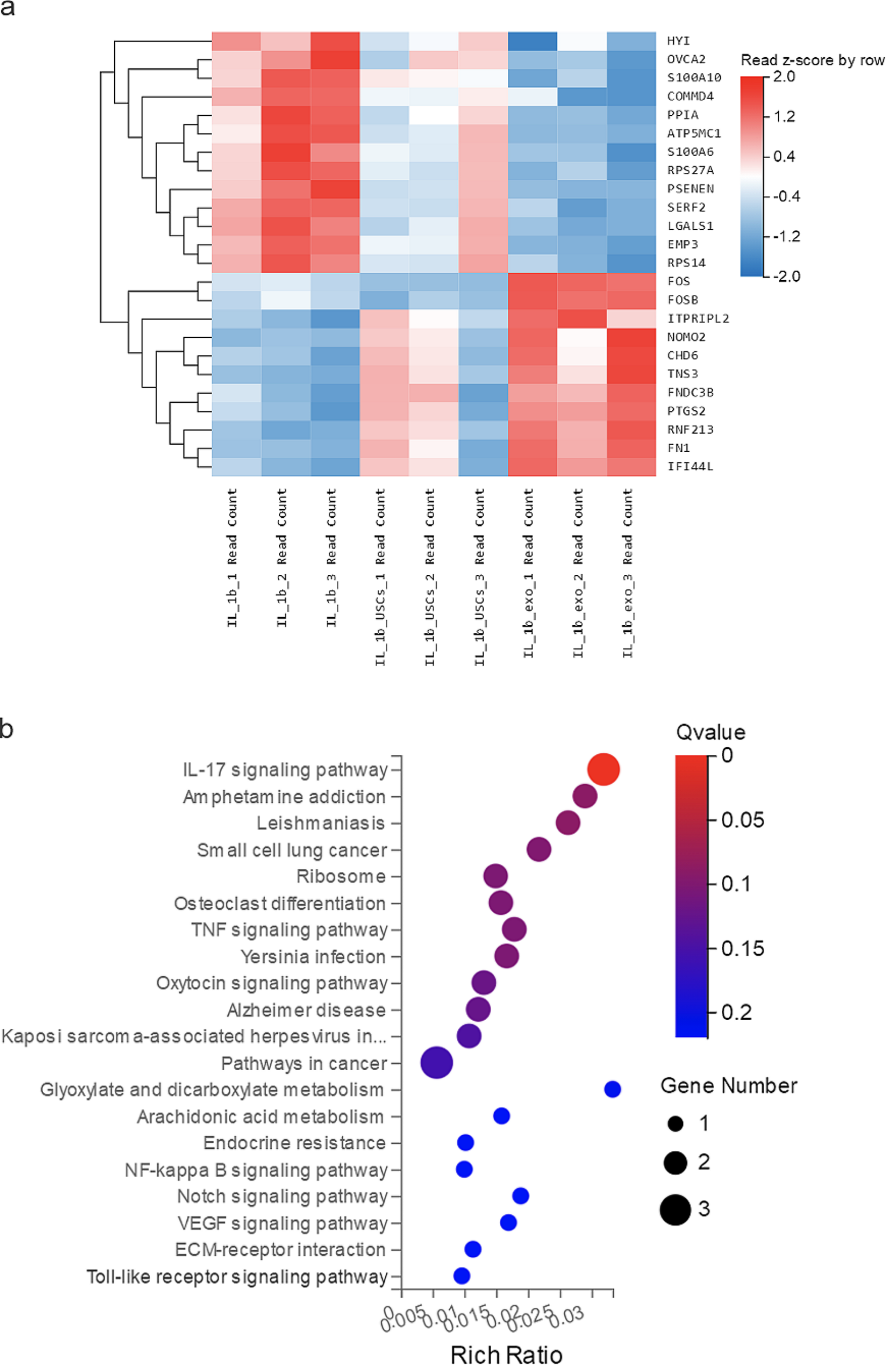
Whole-transcriptome sequencing analysis of IL-1β-induced NPCs under USC or exosomes intervention. (**a**) Heatmap of the top DEGs simultaneously significantly upregulated or downregulated in NPCs following IL-1β stimulation or IL-1β + USCs/exos intervention [*P* < 0.05; fold change > 2.00]. (**b**) GO bubble plots of functional pathway enrichment analysis of cell apoptosis–related differentially expressed genes in NPCs following IL-1β stimulation or IL-1β + USCs/exos conditions

NPC RNA, including circRNA, lncRNA, miRNA, and mRNA, was extracted and whole-transcriptome sequencing was performed. Three IL-1β samples, three USCs + IL-1β samples, and three exosomes + IL-1β samples comprised the test dataset for differential analysis. DEG and GO enrichment analyses were performed to screen for significant differential genes and the related signaling pathways involved in NPC degeneration. Differential genes were analyzed using the limma package, and the differential analyses were visualized as heatmaps (Fig. 8). We identified the top upregulated and downregulated genes between the IL-1β and USCs/exosomes + IL-1β samples, which included *FOS*, *FOSB*, and *ITPRIPL2* (upregulated) and *HYI*, *OVCA2*, and *S100A10* (downregulated). GO enrichment analysis was conducted on the DEGs using EnsembleID to create an enrichment pathway list, and the pathways with a *P* value of < 0.05 were identified as significantly enriched pathways. Among the screened pathways, inflammatory pathways (IL-17 and TNF-α signaling pathways), immune-related pathways (Toll-like receptor pathways), and transcription-related pathways (NF-kB and Notch signaling pathways) were significantly enriched, indicating that the modulation of exosomes or USCs on degenerative NPCs is closely related to cellular inflammation and the intracellular process of transcription.

## Discussion

### Mechanisms underlying inflammatory factor IL-1β-induced NPC degeneration

IVDD is a multifaceted chronic process that alters the structure and function of the IVD. However, the pathophysiology of degeneration is not fully understood. IL-1 is a potent inflammatory and immune-amplifying cytokine mainly produced by macrophages during defense responses. In mammals, IL-1 is a superfamily comprising 11 structurally similar proteins, all of which are involved in inflammation or immunoregulation, primarily through binding to specific receptors on the plasma membrane of target cells. Accumulating data have implied that inflammatory cytokines, especially IL-1β produced by NPCs within the IVD during degeneration, are responsible for stromal degeneration and lower-back pain [30]. In addition, IL-1β reportedly regulates a plethora of events associated with IVD regression, including increased matrix degeneration, decreased matrix synthesis, increased proinflammatory cytokine production, and increased neurotrophic and angiogenic factor production [31–33]. Furthermore, IL-1 receptor antagonist–deficient mice exhibit spontaneous disk degeneration, which, alongside increased risk of lower-back pain in patients with IL-1β polymorphism, suggests a major role of IL-1β in the pathogenesis of disk degeneration [34, 35].

### Apoptotic pathways in which exosomes prevent NPC degeneration

Inflammatory cytokines such as IL-1β or TNF-α are associated with IVDD [36]. IL-1β induces excessive NPC apoptosis. Regulating apoptosis is of considerable significance for treating IL-1β-induced NPC apoptosis. Apoptosis may be involved in the pathophysiology of IVDD, suggesting its importance for IVDD [37]. Apoptosis reportedly reduces the activity of NPCs and changes in the synthesis and composition of the ECM, further contributing to the IVDD pathology. Mitochondrial dysfunction caused by oxidative stress, including a decrease in Bcl-2 levels and release of Bax, triggers caspase initiation, leading to apoptosis [38]. Previous studies have found that pretreatment with spermidine significantly decreased Bax and cleaved caspase-3 levels, while increasing Bcl-2 levels in oxidatively stressed NPCs [39].

### Exosome treatment may be a potential clinical strategy for IVDD

Because NPC degeneration is considered to represent a key initiation site of IVDD, cell therapy specifically targeting NPC recovery has become a potential strategy [5, 29, 40]. Exosomes play a mediating role in immune regulation by eliciting positive and negative immune responses, including tolerance and escape. Individually, exosomes can act as immunomodulators by modulating immune activation, antigen presentation, suppression, and surveillance [41]. However, the exact mechanisms of many of these actions are not fully understood. Several studies have begun to reveal how these vesicles play a necessary and often critical role in initiating various immune responses. Inflammatory responses are often signaled by exosomes, implying that these vesicles are central to various pathological conditions, including cancer, diabetes, obesity, and neurodegenerative diseases. Currently, to the best of our knowledge, there are no approved pharmaceutical interventions or therapies that can prevent the progressive IVDD; however, regenerative strategies aimed at modifying the disease are emerging [42]. New treatments for IVD disease are also emerging; however, considerable challenges remain to be solved across the translational spectrum, from understanding the disease mechanism to useful interpretation for clinical trials, making it difficult to reach a widely accepted consensus [43, 44]. In addition to the incomplete understanding regarding the mechanisms of IVDD, potential challenges include a lack of standardized methods for preclinical testing; an apparent lack of cohesion of tested cell types, tissue sources, expansion conditions, and doses in the context of cell therapy; a lack of information regarding clinical trial guidelines for included disease classification and patient stratification; and incomplete understanding of the mechanisms supporting cell delivery in response to therapy [11].

## Conclusions

Compared with traditional polymeric carriers, exosomes exhibit enhanced biocompatibility, excellent payload capacity, and reduced immunogenicity, and thus, demonstrate great potential as drug-delivery vehicles. Herein, our experiments revealed that IL-1β significantly downregulated NPC viability to induce NPC degeneration while modulating the expression levels of SOX-9, collagen II, and aggrecan. USCs-exo can rescue IL-1β-induced NPC degeneration and restore the expression levels of SOX-9, collagen II, and aggrecan. Thus, USCs-exo can prevent NPCs from degeneration following IL-1β stimulation and can aid the development of a potential treatment strategy for IVDD.

### Electronic supplementary material

Below is the link to the electronic supplementary material.

Supplementary Material 1

Supplementary Material 2

Supplementary Material 3

## Data Availability

The raw sequence data reported in this paper have been deposited in the Genome Sequence Archive (Genomics, Proteomics & Bioinformatics 2021) in National Genomics Data Center (Nucleic Acids Res 2022), China National Center for Bioinformation / Beijing Institute of Genomics, Chinese Academy of Sciences (GSA-Human: HRA007018) that are publicly accessible at https://ngdc.cncb.ac.cn/gsa-human/browse/HRA007018.

## Abbreviations

AF: Annulus fibrosus
BCA: Bicinchoninic acid
ECM: Extracellular matrix
ER: Endoplasmic reticulum
EVs: Extracellular vesicle
IDD: Intervertebral disc degeneration
IL-1β: Interleukin-1β
IL-6: Interleukin-6
IVD: Intervertebral disc
IVDD: Intervertebral disc degeneration
MSC: Mesenchymal stroma cells
NPC: Nucleus pulposus cell
PMSF: Phenylmethanesulfonyl fluoride
TNF-α: Tumor necrosis factor-α
USCs-exo: Exosomes from USCs
USCs: Urine-derived stem cell
