# Supplementary figures and images for: Exosomes derived from human urine–derived stem cells ameliorate IL-1β-induced intervertebral disk degeneration
Source: BMC Musculoskelet Disord. 2024 Jul 12;25:537. doi: 10.1186/s12891-024-07636-2 (PMC11241922; doi:10.1186/s12891-024-07636-2)

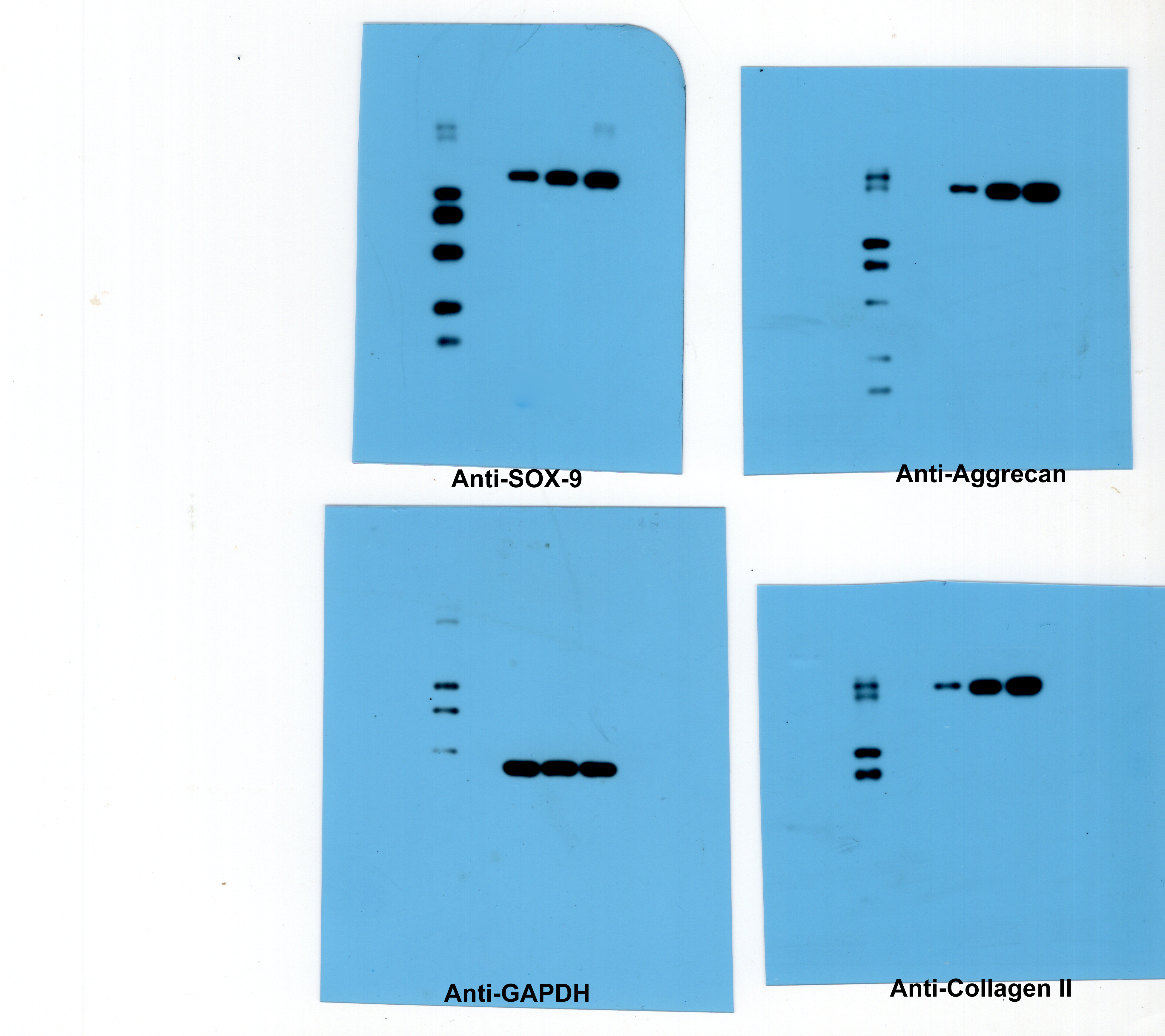

Supplement: Supplementary file 1 — Supplementary Material 1 [file 12891_2024_7636_MOESM1_ESM.png]

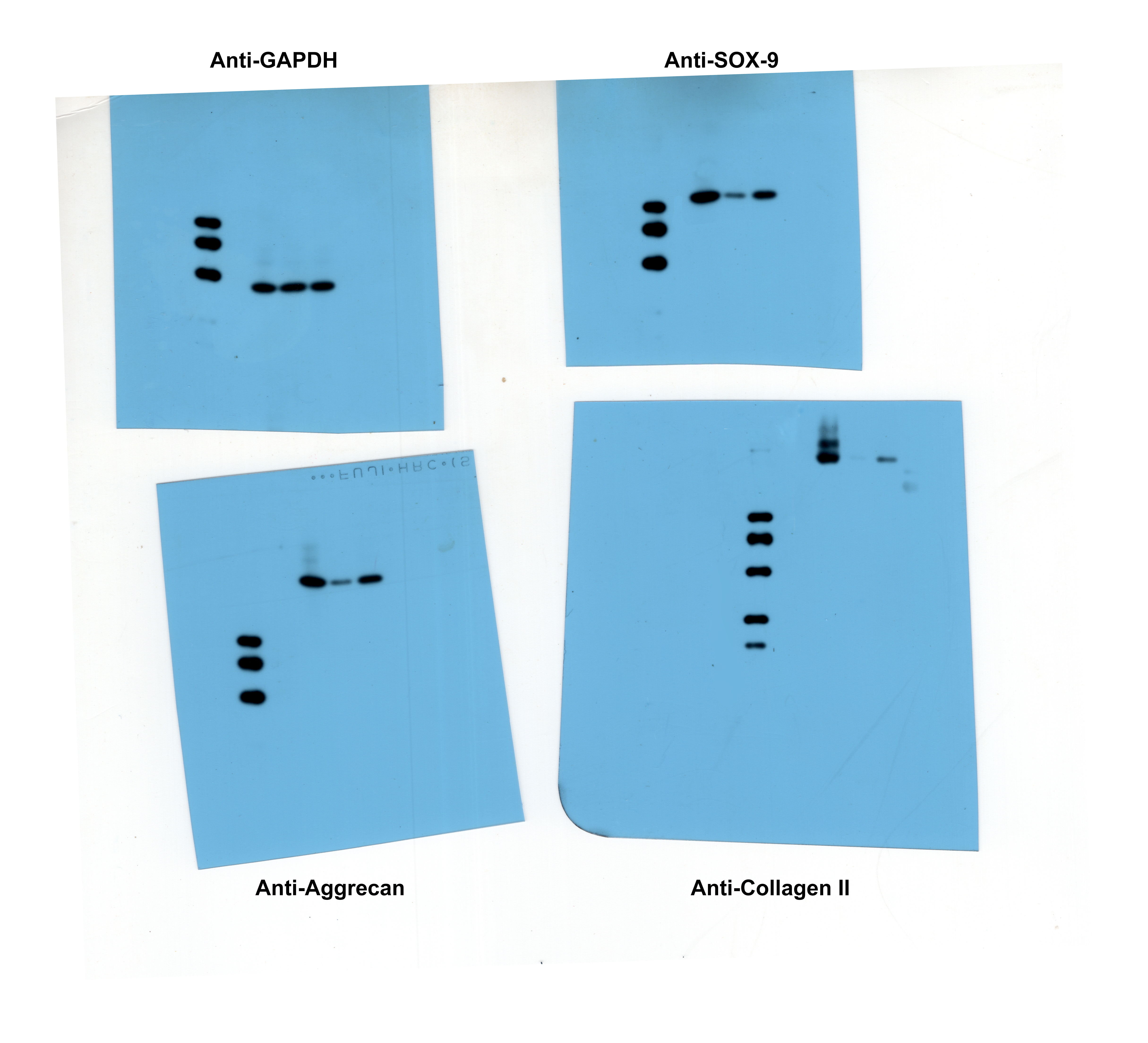

Supplement: Supplementary file 2 — Supplementary Material 2 [file 12891_2024_7636_MOESM2_ESM.png]

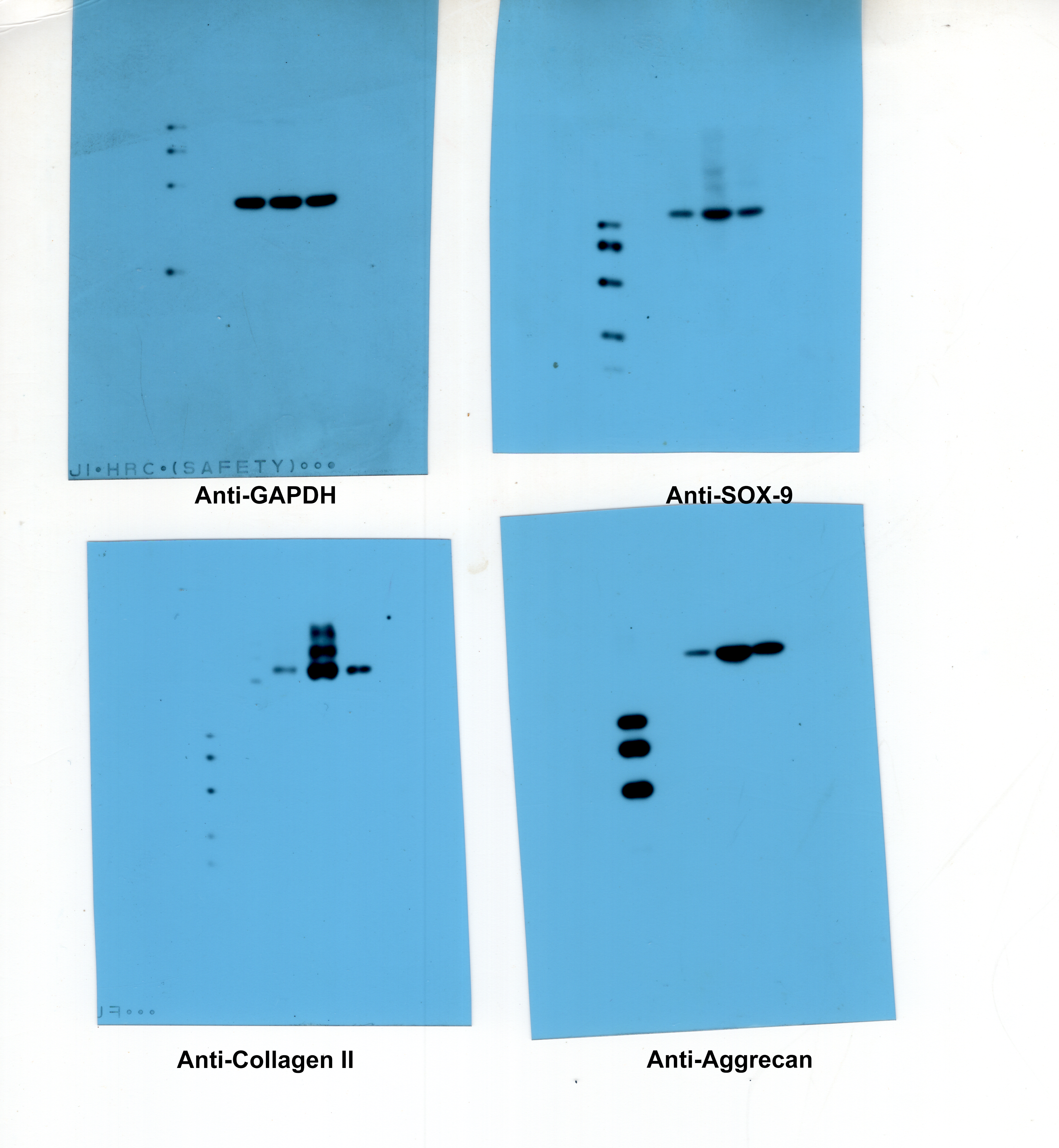

Supplement: Supplementary file 3 — Supplementary Material 3 [file 12891_2024_7636_MOESM3_ESM.png]
